# Report on Surgery

**Published:** 1876-08

**Authors:** Christopher Johnston


AMERICAN JOURNAL

OF

DENTAL SCIENCE.

Vol. X. THIRD SERIES—AUGUST, 1876. No. 4.

### ARTICLE I.

### *Report on Surgery.*

BY PROF. CHRISTOPHER JOHNSTON, M. D.

Read before the Medical and Chirurgical Faculty of Maryland.

*Mr. President and Fellow-Members:*

Guided by the resolution of Dr. John Uhler, offered and passed at our last annual session, your sectionists have sought to confine themselves to the state and progress of Surgery at home, rather than to expatiate upon the art and science abroad.

It has been usual for the Chairman of any section to be allowed or required to make up the report to which his colleagues were supposed or expected to contribute; and this aid was often loyally given by them and accepted by the reporter, although the latter was more frequently suffered to complete his task without the assistance which his co-sectionists might have afforded. With these familiar facts before us, a new departur was resolved upon, the most striking feature of which was the active co-operation of all the several members of the Section, to each of whom was delegated a part of the great subject, with the certainty of his public responsibility for its treatment as over his own name. Accordingly, the Report on Surgery will embrace a short general report by the Chairman, and accessory reports by his colleagues.

In reviewing the career of the Surgeons of our City and State during the year that has gone, but few startling novelties arrested the attention of your sectionists; but everywhere it has been found that a most intelligent zeal has animated the Surgeons, and the boast may be fairly made that surgery has gained materially, although rather by the perfecting of methods already acquired than by the creation of new ones, or the offering of untried speculations. And it will therefore appear that Prof. Erich and Prof. Alan P. Smith's trials of the old method of relieving strangulated hernia by postural taxis find favor with the profession; that the aspirator enjoys a wider range in its sphere of usefulness; that tinnitus aurium has been acceptably explained and its remedy proposed by Dr. Samuel Theobald; and that antiseptic measures as handmaidens of surgical operations obscure or altogether occult the large figures of the ancient death-rate.

There is one matter which your reporter would bring into prominent notice, both from the immense advantages which accrue from it, and because it has always done good service, and this is *Urinometry,* which your sectionists are glad to assure you is practised with increased zeal by all the advanced members of the profession. Time was when great and small surgical operations were undertaken without regard to the condition of the urinary function. Operations of election were attempted without accurate knowledge of the state of the patient's system, and operations of necessity done without investigation as to the quality of the renal secretion; in both sets of cases the surgeon ran the risk of compromising the work which he performed, of jeopardizing or abbreviating the life of the patient, and of meeting with a flat contradiction to the favorable assurances made in his prognosis.

It is, as you are aware, an axiom in surgery that "surgical procedures should not be instituted in notoriously hopeless cases, even with the view of giving the patient what is called the last chance" (Erichsen's Surgery, vol. i.) Under this rule, phthisical patients, and those wretchedly and conspicuously worn by disease, very frequently escape the operating table and its consequences; but not so often the patients laboring under Bright's Disease at a period in its history when the fatal issue is absolutely determined, but when it has, as yet, expressed itself outwardly by but few and unattractive symptoms. To ferret out the covert and lurking danger is becoming, or may be said to have already become, the indispensable preliminary to forming judgment as to the surgical fate of each particular living subject who offers himself before the scalpel, the catling or the gorget. Consequently, if the disease of Bright be found to exist, either milder measures are adopted than might otherwise be sufficient in such case, or, if the proposed operation be one of election, the surgeon may shrink from tempting Providence. Heat, nitric acid and the microscope command the ghostly albumen and weird casts to appear, and they give distinct utterance to the sixth commandment.

If your sectionists had thought themselves permitted to trace the record of surgery in remote lands as well as in our own, of holding up the works of American medical genius everywhere or anywhere, their task would have been more arduous. Not that zeal, knowledge or great ability is lacking in our own honored State, for of all of these the proceedings of our now numerous medical societies of great repute give ample testimony; but because the field being smaller, the gleanings are rarer. It is, however, proposed to offer a few surgical cases in evidence of the spirit which animates the cheirergonists of the profession in Maryland.

The first is a case of transfusion of blood recently done in this city by Professor Frank Donaldson, assisted by Dr. H. P. C. Wilson, and for the history of which, drawn up by himself, your section is indebted to the courtesy of the operator.

MARCH 31ST, 1876.

Mrs. A. B.——, æt. 39 years, a patient of Dr. H. P. C. Wilson, had been suffering over ten years with severe uterine hemorrhages caused by the presence of five fibroid tumors in the walls of the uterus. She had several times during the past three years been near death from the great loss of blood, notwithstandlng the use of the strongest styptics and solutions of iodine. When seen in consultation on the 30th of March she was almost ex-sanguined; was lying in a semi-conscious condition on her back. Her complexion was a very light straw color, and her pulse very feeble, and 126 in a minute. She had been loosing blood for five or six days. She would swallow fluids only in small quantities when urged to do so. She would recognize the members of her family, but could not converse with them. Her brain appeared to us to be in the condition described by Benj. W. Richardson in his experiments in injecting water into the circulation.

It was determined in consultation to try transfusion. To effect this we borrowed from Dr. Jno. Morris his instrument, the *Collin Model*, which had been presented by M. le Prof. Behier to the Academy of Medicine in 1874. This instrument consists of a metallic bowl, at the bottom of which is attached a glass syringe, and a fine tubing ending in a small trocar and canula. Blood was drawn from the arm of a healthy young man into the bowl, which had been previously heated by being plunged into hot water. As the blood was flowing into the bowl, the piston of the syringe was drawn out and the blood flowed into it. It was then pushed through the tubing into the canula, which had been previously inserted into the vein of the patient. Thus the blood was slowly introduced. When *about six ounces* had entered the vein, the patient threw her head back and the heart beat convulsively. We concluded that we had introduced as much as the heart, so long accustomed to her thin watery blood, would bear, and instantly stopped the operation.

The patient was in a few seconds revived. The pulse was manifestly stronger and the color of the patient had improved. Her mind was clearer and her sensibility was more acute. She complained of a violent pain in her back, from which she had been suffering previously. She became very restless, and suffered so acutely with her back that it was determined to give her a hypodermic of morphia. This soothed her and she slept for six or seven hours profoundly. After the effects of the morphia wore off she lived for two days, gradually sinking.

The transfusion was only temporary in its effects. The long continuance of the chronic disease and the draining of her system of blood for so many years prevented any more lasting effects, yet, as a last resort, in a desperate case it seemed justifiable, and it produced no bad results.

Collin's instrument is a perfectly safe one, both as regards the person from whom the blood is drawn and the patient. In the former it is simply connection which is performed. In the latter there is no danger from the introduction of air, which is well guarded against, nor is there any risk of forcing in coagula of blood if care is taken to keep up the temperature of the bowl. I would suggest an improvement to secure the bowl being kept warm, by rendering its sides hollow and putting hot water into the cavity.

Transfusion is not necessarily confined in its employment to blood, as is shown by a case of transfusion of lamb's blood in a case of consumption, and given in the *Boston Med. and Surg. Journal* for January 14, 1875, without commendation. Salines also have been employed in cholera attended with great prostration after exhausting rice-water discharges; and milk, or milk and defibrinated blood, more recently by Mr. Wagstaffe. "Milk alone was employed twenty years ago," says Prof. T. Gaillard Thomas, "by Dr. Edward M. Hodder, of Toronto, Canada. In 185[illegible] Dr. Hodder injected this fluid into the veins of three patients moribund from Asiatic cholera, which was at that time epidemic in Canada. In a communication from him," continues our author, "he informs me that he injected as much as fourteen ounces at one sitting; that no alarming symptoms occurred; that good results manifested themselves from the first, and that two recoveries had taken place in patients who appeared moribund when the operation was resorted to. He was encouraged to try the method from the fact that Donne injected milk into the veins of dogs and rabbits without injury to them."

In the *American Journal of Med. Sciences* for January of this year, Prof. T. Gaillard Thomas himself gives to the world an account of a brilliant success attending the transfusion of milk in a patient upon whom, four days before, he had performed double ovariotomy, for the reason that after two severe uterine hemorrhages, and as "the patient could take very little food without vomiting, she appeared to be dying from sheer exhaustion, the result of previous enfeeblement by the disease, and more recent starvation and loss of blood." After three ounces of warm, perfectly fresh milk had been injected, the pulse became so rapid that it could hardly be detected. The patient declared that she felt as if her head would burst, and seemed greatly overcome. The operation, however, was continued slowly, until eight and one half ounces had been reached, and she was left perfectly quiet. In an hour from this time she had a decided rigor, the pulse was found beating between 150 and 160 to the minute, and the temperature rose to 105°. This high rate of temperature, however, soon passed off, and towards midnight the patient fell into a quiet sleep, from which she did not awake until morning. Six weeks after the operation the patient was entirely well."

A consideration of all the circumstances in which transfusion is demanded, leads us to the belief that blood will not always satisfy the demand. In cases of sudden and prostrating hemorrhage, traumatic or other, transfusion of human blood is most urgently insisted upon by many authors, and by none with more earnestness than the venerable Professor Samuel D. Gross; but in chronic exhaustion, when the natural avenues for nutriment from without are closed, or especially when persistent emesis and diarrhœa are rapidly wasting the patient, some fluid other than blood might or ought to find more favor and be more apposite; and milk might better supply the want of the system.

It is on all sides conceded that the apparatus used should be free from value; indeed so objectionable were these to that eminent surgeon Mr. Wagstaffe, that in the cases already referred to he adopted the apparatus suggested by Dr. Hamilton, of Ayr, which consisted of a funnel with about two feet of elastic tubing attached to it; when the funnel is elevated, the weight of the fluid employed forces it with equal, gentle and desired pressure into the veins.

With regard to the condition of the blood to be transfused, writers are not in accord; some, as Panum of Copenhagen, advocating defibrination, while perhaps the majority, or at the least the most modern men, as Prof. Gross, prefer the direct transit. To what extent this difference of opinion may go is not, however, greatly important; and the solution of many problems with regard to transfusion is reserved, possibly for men of our own day.

From transfusion we turn to that unusual vice of development, exstrophy of the bladder, a case of which, during the last winter, offered itself for operation to one of your sectionists. The malformation is that in which, from deficiency in the abdominal wall and anterior paries of the bladder, the posterior wall of this viscus—appearing to be turned or twisted out of place—is let into the gap, appears uncovered, with the orifices of the ureters exposed, and its mucous membrane continued forward, in the male, into the urethral groove in the incomplete penis. In the female the condition is essentially the same, difference in sex being observed ; in both the public bodies are wanting, and inguinal hernia exists on one or both sides. And this, as you are aware, is only one of the many deviations from the normal which have occurred, representing all allantoic aberrations from the simply pervious urachus, to that malformation in which the bladder, being undeveloped, the ureters open directly into the rectum, or are continuous with the urethra.

These various abnormalities have, of course, an interest in themselves; but for the surgeon they acquire a value from the fact that some of them have suggested to him ways for the possible relief of exstrophy. Finally, while the extroverted bladder is in general of unfrequent occurrence, it is exceptionally rare in the female; thus Prof. Gross in 1872 says that of 16 cases which came under his notice, but two were females. Of 9 cases observed by Mr McWhinnie, 7 were males; and Mr. John Wood has met with upwards of 20 cases, of which only two were females. And your reporter has seen 4 cases, of which one occurred in the woman.

All operative procedures designed to alleviate the condition of patients afflicted with exstrophy of the bladder may be ranged under two captions:—1st. That one having for its object the establishment of a *fistulous communication* between the ureters and rectum, as practised long ago by Simon, of St. Thomas' Hospital, and by Messrs. Lloyd and Athol Johnson; and three varieties of plastic operation, *one* by *flap*, derived from the umbilical region, the groins or the scrotum, as done by Richard, Pancoast, Maury, and Prof. Wood of King's College London; *another* by *gliding* towards the median line integuments dissected up on either side, after the manner of Barker of Melbourne; and finally, *a third* or mixed operation, in which both flap and gliding methods were employed by Ayers, of Brooklyn, and Mr. Holmes. The operation presently to be described is to be ranged in the first category.

In the clinic of your reporter there appeared, in the month of December last, and in the same hour, two males, both subjects of vesical exstrophic deformity, the one a man of 44 years, the other a boy of eight. In both the umbilicus was wanting, as were the public bodies; a deeply red oveal area of oozing mucous membrane in the supra-pubic region represented the bladder; two small pores at its lower part were the opening of the ureters; the penis was as if split horizontally, and the urethra reduced to an uncovered groove continuous with the vesical membrane. Below was a small scrotum provided with diminutive testicles; while above, on either side, an inguinal hernia swelled with every effort of the subject. And the red surface was moist with mucus, and the penis, scrotum and thighs were incessantly irrigated with urine, the odor of which was extremely disagreeable.

After the lecture, the boy, who had before been shy and timid, announced his determination to undergo an operation, for, said he, "I would rather die than grow up like that man." On the 22d January, 1876, the operation was done. The red bladder measured $2\frac{1}{2}$ inches in width by $2\frac{1}{2}$ inches in height, with ample space above to the xiphoid cartilage. The boy was spare, but apparently healthful. After etherization, an operation, essentially that of Wood, was undertaken. A great umbilical flap was brought down over the bladder, the skin surface lying under, and this was covered by two wing flaps, one from either groin, which met in the middle line, and were secured to the first oblong flap. The space left by the umbilical flap was almost effaced by drawing together the integuments of opposite sides, and which had been dissected up; while the spaces left by the wing flaps were obliterated by a gliding of the skin which lay beyond the groins, made free underneath, and attached to the outer border of the wing flaps, now fixed in their new situation by silver and iron wire. As tension strained the lateral flaps, a crescentic incision was made beyond each groin to reduce it. After the operation the little patient suffered exceedingly, principally on account of scalding by the urine; but in about five weeks he was able to return home greatly benefitted, the operation having proved a success, perfect with the exception of a small loss of substance at the lower edge of the umbilical flap. In its present condition the child will remain until cicatrization shall have completed its contraction, whereupon a second operation will be attempted, having for its object the formation of a sort of roof for his urethral groove. As things are, however, the urine escapes over and around the penis only, and he is able to wear advantageously a railroad urinal.

In connection with the treatment of fractures your sectionists are happy to notice the "Anterior Extension Splint" of Dr. G. E. Porter, of Lonaconing, Maryland, an account of which, and of six cases of fracture of the femur treated with the splint, appeared in the *Medical and Surgical Reporter* of Philadelphia for March, 1876. The first of these cases bear date June 11th, 1874, and the last September, 1875. From the journal referred to we borrow the drawing of the apparatus and the description of the author.

Dr. Porter says, on page 224, "The drawing will I think make the descriptive parts of the paper clear." "It must be borne in mind," continues the writer, "that the splint is intended to apply almost wholly to fracture of the femur in adults. At the same time, I know of no apparatus equally well adapted to the treatment of compound and gunshot fractures, to disease of the articulations requiring extension, and to cases of subperiosteal excision of the bones of the lower extremities.

"The splint is made of stout wire; the number five wire of the shops will be found large enough for adults, the number six for children. From its pelvic extremity to the bend at the instep it is two inches wide; from the latter point it gradually widens, until where it bends over the toes and passes down vertically in front of the plantar surface it is five inches wide. The cross wires are arranged with about six inches between them, and securely soldered to the side wires. The splint is bent at the groin and the instep to about the same angles as in the old anterior splint. There is a slight bend at the knee, to clear the patella, and for other obvious reasons. It should be of sufficient length to reach the anterior superior iliac spine. What may be called the plantar part should extend a little below the posterior horizontal line of the heel. The curvatures of the foot portion of the splint are to be made with a view of giving the greatest possible firmness. The apparatus for suspension employed by Prof. N. R. Smith is the best that can be devised, if we add a second short cord for the immediate support of the splint. Four points of support afford increased steadiness and firmness to the appliance.

"Instead of the roller, I used the many-tailed bandage, in two pieces: one piece long enough to reach from the knee to the perineum. The tails, two and a half inches wide, should be well cut down, leaving a narrow isthmus along the posterior mesian line of the limb, and long enough to embrace the limb and pin over the side-wires, with three or four inches to spare. There should be two strips of adhesive plaster, each twenty-four inches long, four inches wide at the knee, and three at the foot end. A paper of pins and some cotton batting must be at hand. *The many tailed pieces must always be made of stout, unstretching brown drilling.* This is an essential point. The adhesive strips are pinned over the plantar side-wires, and in a line with the axis of the limb."

As a result of treatment in the six cases reported, Dr. Porter claims that there was *no shortening* in two cases, shortening to the extent $\frac{1}{4}$ inch in two, $\frac{3}{4}$ inch in one, and $\frac{1}{2}$ inch in one case. An examination of the instrument and of its mode of application will at once suggest Prof. N. R. Smith's method of obtaining extension through adhesive straps adopted three years ago; although it must be admitted that many of the details of Dr. Porter's apparatus are certainly novel, and perhaps advantageous features, as for example the narrowness of the space between the wires of the splint frame, the peculiar bending of foot-part, and the mode of adjustment of the adhesive straps.

Before leaving the subject of fractures, your section desires to call attention to Dr. Porter's wire splint for the forearm. It is here presented, and consists of a wire frame wider than the forearm, with a short L deviation at the lower part to accommodate the hand as far as the metacarpo-phalangeal articulations. Usually splints of this kind are sold in pairs, a right and a left hand one, but either may be employed with excellent effect if applied on the opposite forearm in case of fracture of the ulna alone. Before being used the splint must be tightly wrapped with bandage, which then forms a solid but not rigid bed for the injured member, and may be cushioned according to the kind of fracture. It is a light and elegant contrivance; and from experience your sectionists can speak of it in terms of high commendation.

In concluding this report, your section cannot refrain from insisting upon immense benefits which have accrued to surgery as well as to medicine from the enlightened efforts of the sanitarium. Not only are ligatures and sutures rendered antiseptic, but surgical instruments are themselves disinfected, so that the lament of surgeons of a former day is no longer heard in our midst. Cleanliness of persons and things is enjoined in hospitals; foci of disease are destroyed and not allowed to reform; the odorless excavating apparatus is made to carry away, even under a garish sun, accumulations of necessary household filth and possible contamination. Cities, nay districts and countries, are instructed in the art of destroying the destroyer, and made to recognize the power of human science, and admit it in the diminished death rate and the increasad average duration of human life.

Formerly, hospitals shared the benefits of disinfection and ventilation to a limited extent only, because great principles could be applied but partially in structures erected after ancient plans, in which both surgeon and patient braved the danger alike. But now-a-days, hospitals are planned especially with a view to afford, not only a great number of cubic feet to each patient, but also to secure the constant withdrawal of effete air, and its replacement with other that is fresh and uncontaminated.

Among the men who, having the *means* to carry good intentions into effect, manifest their zeal and intelligence in advancing the congenial interests of humanity and promoting the benevolent designs of that princely giver, the late Johns Hopkins, must be mentioned with respect and gratitude the able board of Trustees to whom are committed the erection of wards for the Hospital connected with the University. All successes in existing institutions are or have been scrutinized, all failures as faithfully studied; the wisdom of the civil and of the military surgeon and physician has been drawn upon, and the first fruits of such enterprise are a series of plans as modern as they are advanced. Still the architect and the physician mutually strengthen and aid one another; and these plans must yet be subjected to the ordeal of a triple inspection.

Mr. Niernsee, a distinguished architect of Baltimore, in reviewing, at the request of the trustees, the plans alluded to, became sensible of the necessity for the renewal of air, and of the dangers incurred by patients from lateral dissemination of miasms; and he bethought him of the admirable and thorough system of ventilation invented, and long since put in practice at his residence, by Mr. Thomas Winans. This system, assuming that a large number of cubic feet shall have been allotted to each of the possible individuals occupying every apartment, involves four conditions: *first*, the existence beneath the floors of air-chambers in which fresh atmospheric air shall be tempered thermometrically and hygroscopically according to the season; *second* the perforation of the floors by an immense number of nearly half-inch holes; *third*, free valvulated openings in the walls near the ceiling, or in the vault; and, *fourth*, the powerful suction of a tall aspirating chimney, into which open all the converging vomicæ of foul air, which is wafted aloft along with the smoke of furnace and kitchen in winter, and of especially lighted fires in the warm months. Indeed, in the hottest and most breezeless day of the heated term, a cooling and refreshing zephyr can be and is perpetually maintained.

As by this arrangement the air set in motion is hurried upward, it follows that pernicious exhaltations must share the same fate. And another consequence, anticipated by the ingenious inventor, offers striking advantages, which is, that in winter and the cooler season the *lower strata* of air are *warmer* than the *upper ones*, of which Mr. Winans keeps the demonstration before him in a series of thermometers fixed at different heights. Now Mr. Niernsee proposes to make available for the Johns Hopkins Hospital the Winans successful system of ventilation—a part of which in the form of water-action valves, is actually in operation in the little tower of the Academy of Music. Mr. Niernsee suggests that the plan be adopted; but that the floors be perforated under each bed only, and that every sub-pedal air-chamber, with its entering and emergent drafts, be under separate control. The reservation is, without doubt, a wise one, for sufficient ventilation could be obtained in the way indicated, and, of course, leaving the treadway and interlectular spaces imperforate. To reproduce Mr. Winans' plan exactly would require an area of the perforations to equal 1-10th of the whole area of the floor, as Mr. Winans' saloon floor, 60 feet by 30=1800 square feet, is perforated by 180,000 holes nearly half an inch in diameter, equal in area to 180 square feet, or one tenth of the superficial measure

All of which is respectfully submitted.

